# Influence of Bridging Stent Graft Implantation into the Renal Artery during Complex Endovascular Aortic Procedures on the Renal Resistance Index

**DOI:** 10.3390/diagnostics14171860

**Published:** 2024-08-26

**Authors:** Daniela Reitnauer, Kerstin Stoklasa, Philip Dueppers, Benedikt Reutersberg, Alexander Zimmermann, Thomas H. W. Stadlbauer

**Affiliations:** 1 Department of Vascular Surgery, University Hospital Zurich, University of Zurich, 8091 Zurich, Switzerland; daniela_reitnauer@icloud.com (D.R.); k.stoklasa@web.de (K.S.); dr.dueppers@gmail.com (P.D.); benedikt.reutersberg@usz.ch (B.R.); thomas.stadlbauer@usz.ch (T.H.W.S.); 2 Department of Vascular Surgery, Kantonsspital St. Gallen, 9007 St. Gallen, Switzerland

**Keywords:** renal resistance index, renal artery, vascular ultrasound, abdominal aortic aneurysm, endovascular aneurysm repair, complex EVAR, FEVAR, BEVAR, bridging stent graft

## Abstract

Comparative sonographic examination of the renal resistance index (RRI) can provide evidence of renal artery stenosis. The extent to which the RRI is changed after stent graft implantation is not known. The aim of this study was to investigate the influence of stent graft implantation into non-diseased renal arteries during endovascular treatment of pararenal aortic aneurysms on the RRI. Sonographic examinations of the kidneys were conducted using a GE ultrasound system. The evaluation was performed according to the European Society for Vascular Surgery (ESVS) 2D standard criteria. RRI values were determined in consecutive patients on the day before and after stent graft implantation and compared for each kidney. A total of 32 consecutive patients (73.9 ± 8.2 years, 5 females, 27 males) were treated with a fenestrated or branched aortic stent graft including bridging stent graft implantations into both renal arteries and received pre- and postinterventional examinations. Sonomorphologically, the examined kidneys were inconspicuous. The arborisation of the renal perfusion was preserved pre- and post-implantation. The RRI did not differ (0.66 ± 0.06 versus 0.67 ± 0.07; *p* = ns). Successful stent graft implantation into non-stenosed renal arteries did not lead to a relevant change in RRI. Therefore, the RRI is a suitable tool for assessing renal perfusion after fenestrated or branched endovascular aortic therapy.

## 1. Introduction

Aortic aneurysms most commonly affect the infrarenal aortic segment. If untreated, an abdominal aortic aneurysm (AAA) can lead to death in the case of rupture [1]. Depending on morphological parameters, comorbidities, and patient preferences, AAA can be treated endovascularly or by open aortic repair (OAR). However, endovascular aortic repair (EVAR) should be considered the preferred treatment modality in elective patients with suitable anatomy and reasonable life expectancy. In addition open repair should be considered in most patients with long life expectancy and absence of relevant comorbidities [2,3].

Compared to standard aortic pathologies with adequate infrarenal neck morphology, the treatment of complex aortic aneurysms (CAA), defined as juxta-, para-, suprarenal, or thoraco–abdominal aortic aneurysms, is more complex and invasive [3,4]. Despite mortality rates up to 15%, the traditional standard of care in these cases is OAR. Due to the highly invasive nature of OAR, endovascular techniques have evolved over the last 20 years to treat CAA with a minimally invasive approach [5,6,7]. These treatment modalities include fenestrated (FEVAR) and branched (BEVAR) stent grafts. The aim of those procedures is to exclude the aneurysmatic segment of the aorta from the arterial circulation while maintaining vital perfusion to the internal organs using bridging stent grafts for the renal and/or visceral arteries. For CAA patients considered high-risk for OAR, several studies have demonstrated promising short and midterm outcomes for F/BEVAR, resulting in a significant increase in F/BEVAR procedures worldwide, even in patients after prior OAR [2,8,9,10,11,12,13]. Due to its high technical complexity, F/BEVAR is associated with multiple challenges. Target artery complications, like perforation, disruption, or dissection are reported in 9% to 19% of cases in two recently published retrospective studies [14,15]. A systematic review published in 2021 indicated a higher rate of target vessel complications in renal arteries (6%) than in visceral arteries (2%) [16]. Therefore, it has been suggested that the detection of bridging stent graft kinks or compressions is mandatory to prevent and identify end-organ malperfusion [17]. Intraoperative digital subtraction angiography (DSA) as the method of choice to detect possible endoleaks and to verify correct stent graft deployment and patency as well as postoperative computed tomography angiography (CTA) as primary imaging modality for follow-up have their limitations and expose the patients to additional radiation and iodine contrast agent [3,18]. 

An alternative for bridging stent graft evaluation is pre- and postoperative color-coded duplex ultrasonography (DUS), which does not include radiation or nephrotoxic contrast agent exposure. One important ultrasound-specific parameter is the renal resistance index (RRI), which is calculated from the difference between the peak-systolic (PSV) and end-diastolic (EDV) blood velocities divided by the PSV [19]. Ultrasound examination of the RRI of both kidneys can provide evidence of renal artery stenosis as long as it is not altered by the bridging stent graft implantation itself [20,21,22].

To our knowledge, there are no data comparing pre- and postinterventional RRI after bridging stent graft implantation. Therefore, the aim of this study is to compare the pre- and postoperative RRI in F/BEVAR, and to evaluate if the RRI can be used as a valid parameter for renal and renal artery assessment after stent graft implantation. 

## 2. Material and Methods

### 2.1. Study Population and Selection Criteria

All patients who were treated with F/BEVAR at a tertiary referral hospital between January 2020 and February 2024 were retrospectively analyzed. The institutional clinical information system (KISIM 5.1.0.3; CISTEC AG, Zurich, Switzerland) was screened for eligible patients and used for the collection of demographic, perioperative, and imaging data. The regional ethics review board provided ethical approval (No. 2024-00581). Patients meeting the following criteria were eligible to be entered into this retrospective analysis: the presence of written consent, a CAA treated with F/BEVAR, and successful bridging stent graft implantation in both renal arteries during the study period. We excluded all patients with parallel grafts, pre-existing known relevant renal artery stenosis (pre-operative CT scan), previous renal artery stenting, or previous parallel graft implantation into the renal artery. Also, patients with perioperative renal artery dissection or occlusion, postoperative severe acute renal failure as well as patients without proper documentation of pre- and/or postoperative RRI measurements (e.g., six RRI/side pre- and postoperative) due to multiple reasons were excluded.

### 2.2. Clinical Data and Surgical Aspects

The following clinical data were evaluated: demographic characteristics, cardiovascular risk profile, clinical presentation, and laboratory results (lipid profile, creatinine/estimated glomerular filtration rate (eGFR), HbA1c). Hypertension was defined according to ESC clinical practice guidelines with an office blood pressure above 140/90 mmHg and/or treatment with antihypertensive drugs [23,24]. Diabetes mellitus was defined by an HbA1c > 6.5% at inpatient admission according to ESC clinical practice guidelines and/or treatment with antidiabetic drugs [25]. Dyslipidaemia was defined according to ESC clinical practice guidelines with LDL cholesterol > 3.0 mmol/L and/or treatment with lipid-lowering medication [24,26]. Pre- and postoperative creatinine and eGFR were extracted from the clinical records. Maximum aneurysm diameter, indications, and surgical details (type of aneurysm, used prosthesis and bridging stent grafts, bridging of visceral arteries) were extracted from the surgical reports. According to current Reporting Standards for F/BEVAR, technical success was defined as successful catheterization and stent graft placement in both renal arteries as intended target vessels of interest. Primary patency was defined by documented stent graft patency in the intraoperative DSA without detection of any kinks, occlusion/ thrombus in the stent graft, or dissection of the target vessel [27].

### 2.3. Perioperative Renal Protection

To prevent contrast-associated acute renal injury, we followed a perioperative regime according to the latest professional society guidelines. This included the identification of patients with already impaired renal function with routine, preoperative control of creatinine and eGFR values, adequate pre- and perioperative hydration, and minimalization of contrast agent exposure. Postoperatively, patients were treated at the intermediate care unit or intensive care unit with monitoring of the diuresis [3,28]. 

### 2.4. Ultrasonography

Ultrasound examinations of the kidneys were conducted routinely using a GE LOGIQ S7 XDclear ultrasound system (GE Medical Systems AG, Glattburg, Switzerland) with a 1–6 Mhz, C1 curved array transducer by two trained investigators (first and senior author). The scans and evaluation were performed according to ESVS standard criteria [3]. The RRI was determined in all consecutive patients on the day before and after bridging stent graft implantation into the renal arteries. Sonomorphologically, the maximal length and diameter of the examined kidneys, as well as the parenchymal margin, were detected. Thereafter, the kidneys were divided into three areas according to the standard protocol as proposed by Radermacher et al. and two RRI values were recorded per area by evaluating intrarenal arterial Doppler signals from segmental arteries [22]. Mean values were calculated and compared for each kidney.

### 2.5. Statistical Analysis

The statistical evaluations were conducted with the StatsDirect statistical software (StatsDirect Ltd., Wirral, UK, Version 1.9.8). For the demographic characteristics (i.e., age at intervention, sex), the cardiovascular risk profile (i.e., arterial hypertension, smoking, diabetes, dyslipidaemia), the maximum aneurysm diameter, length of hospital stay, surgical aspects (type of aneurysm, used prosthesis and bridging stent grafts, bridging of visceral arteries), and the indication (primary or secondary) descriptive statistical analysis were applied. If indicated, these values were expressed as mean ± standard deviation. In addition, subgroup analysis was performed for stent graft diameter and length. The subgroups were defined ≤ and > of the median of the complete cohort. Furthermore, subgroups were defined by performed procedures (FEVAR/BEVAR). The statistical relevance was evaluated with unpaired two-sided *t*-tests in the case of normally distributed parameters (F-test). 

For the sonomorphologic parameters (kidney length, diameter, and parenchymal margin), the pre- and postoperative RRI, as well as creatinine values and eGFR were evaluated with unpaired two-sided *t*-tests in the case of normally distributed parameters (F-test). A *p*-value < 0.05 was considered statistically significant.

## 3. Results

### 3.1. Screening Algorithm

**Figure 1:**
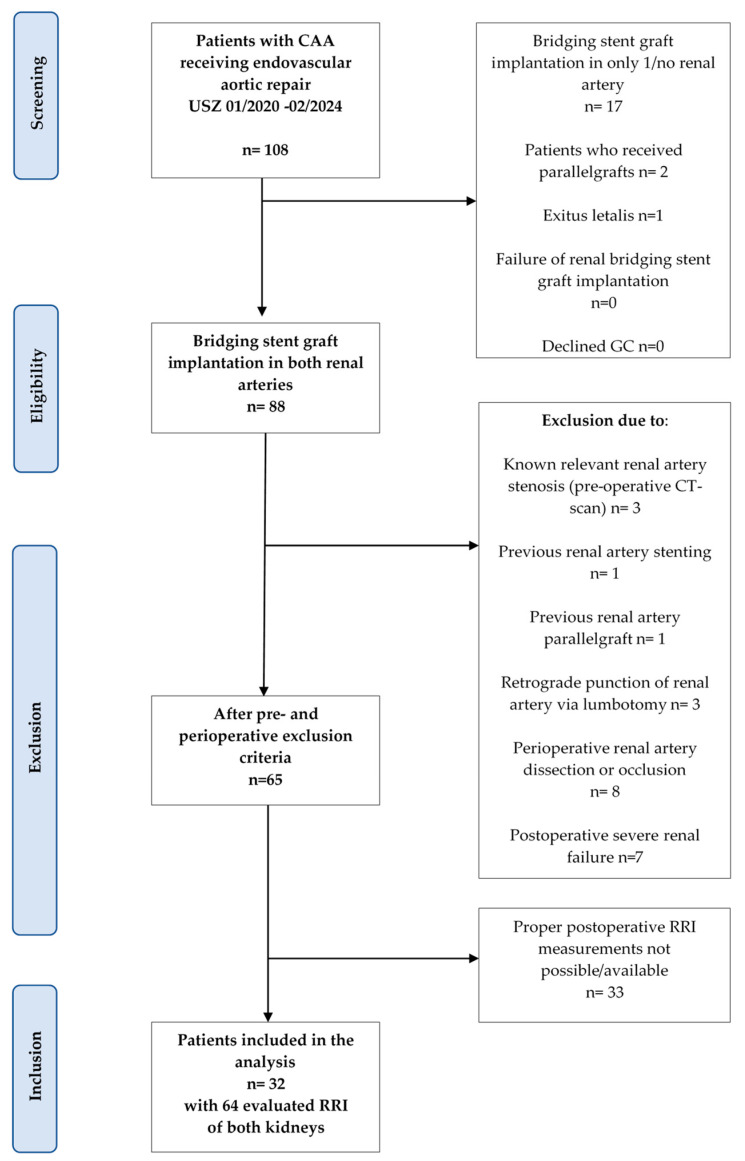
The total dataset contained all hospitalizations in the Department of Vascular Surgery at the University Hospital of Zurich. After screening, 108 patients were detected and from these, 88 were eligible. After the application of pre- and postoperative exclusion criteria, 65 remained. A complete dataset could be evaluated from 32 patients.

A total of 108 patients with fenestrated or branched stent grafts were included in this study (Figure 1). A total of 20 patients were excluded due to omitting bridging stent graft implantation into one or both renal arteries, parallel graft implantation, or perioperative death. Thus, 88 patients with successful bridging stent graft implantation in both renal arteries were identified. Twenty-three patients had to be excluded because of preoperative known renal artery stenosis, previous renal artery stenting or parallel graft implantation, retrograde puncture of the renal artery via lumbotomy, perioperative renal artery dissection or occlusion, and postoperative acute renal failure. Of the remaining 65 patients, 33 patients had to be excluded due to a lack of adequate pre- and postoperative RRI measurements (6 RRI/kidney). Therefore, 32 patients were included in this retrospective analysis with a total number of 64 kidneys studied.

### 3.2. Patient’s Characteristics

The mean age of the 32 studied patients was 73.9 ± 8.2 years. Twenty-seven (84%) were males and five (16%) were females. Thirty patients suffered from arterial hypertension (94%), 31 patients had dyslipidemia (97%), and four patients (12%) suffered from diabetes mellitus. Twenty-five patients (78%) had a history of smoking. The baseline characteristics of the study population are summarized in Table 1.

### 3.3. Anatomic Characterization of the Underlying Pathology

Twenty-two patients (69%) presented with a juxtarenal aneurysm, eight (25%) with a thoraco–abdominal aortic aneurysm (Crawford type I = 2, type II = 3, type IV = 2, type I = 1), and two (6%) with a pararenal aneurysm. The mean preoperative maximum aortic diameter was 62.8 ± 11.2 mm.

### 3.4. Endovascular Treatment Modalities

Nineteen patients (59%) were treated electively with a CAA, and eleven patients (34%) had an elective repair after former EVAR. Two patients (6%) were treated on an urgent basis due to a symptomatic aneurysm. A total of 26 patients (81%) received a FEVAR and six patients (19%) a BEVAR. Further details of the endovascular procedures are displayed in Table 2. 

### 3.5. Bridging Stent Graft Characteristics

A total of 66 bridging stent grafts were implanted in 64 renal arteries. There were two cases with the implantation of two bridging stent grafts in each renal artery. Bridging stent grafts from five different manufacturers were used. Four manufacturers with balloon-expandable bridging stent grafts: 15 Advanta V12^®^ (Getinge AB, Stockholm, Sweden), one BeGraft^®^ (Bentley, Hechingen, Germany), eight E-ventus^®^ (Jotec, Hechingen, Germany) and 42 iCover^®^ (iVascular, Barcelona, Spain), one manufacturer with one self-expanding stent graft: Viabahn^®^ (Gore, Newark, DE, USA). A total of nine bridging stent grafts with a diameter of 8 mm were implanted, one with a diameter of 7 mm and 56 with a diameter of 6 mm. The median diameter was 6 mm. The length of the implanted bridging stent grafts varied between 17 mm and 58 mm with a median length of 27 mm (Table 3). 

### 3.6. Ultrasound Evaluation

**Figure 2:**
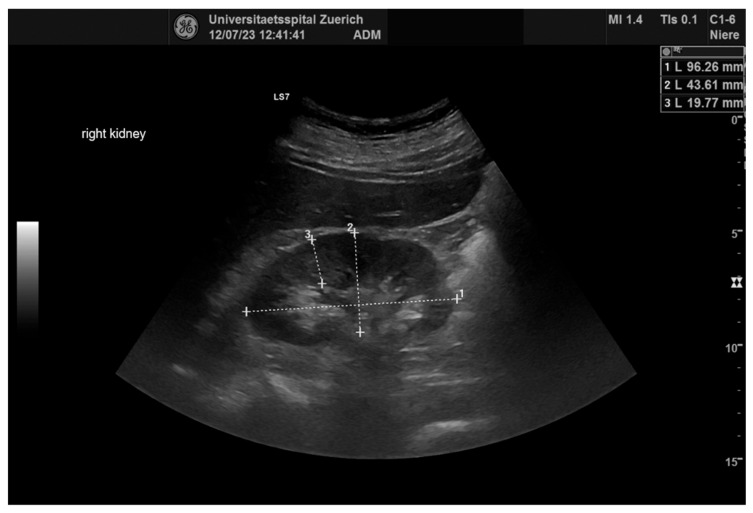
B-mode renal ultrasound right kidney. 1: length of kidney, 2: width of kidney, 3: width of kidney parenchyma.

**Figure 3:**
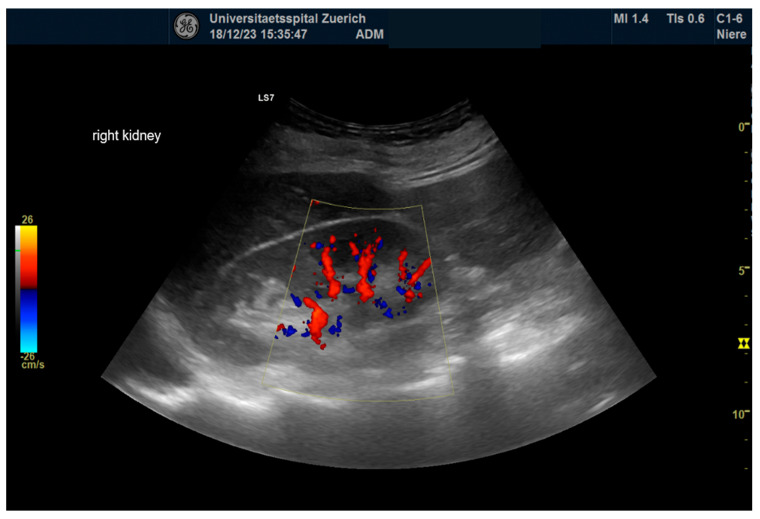
Kidney arborization. Picture of complete arborization of the kidney in duplex mode with “fire and flame” phenomenon.

**Figure 4:**
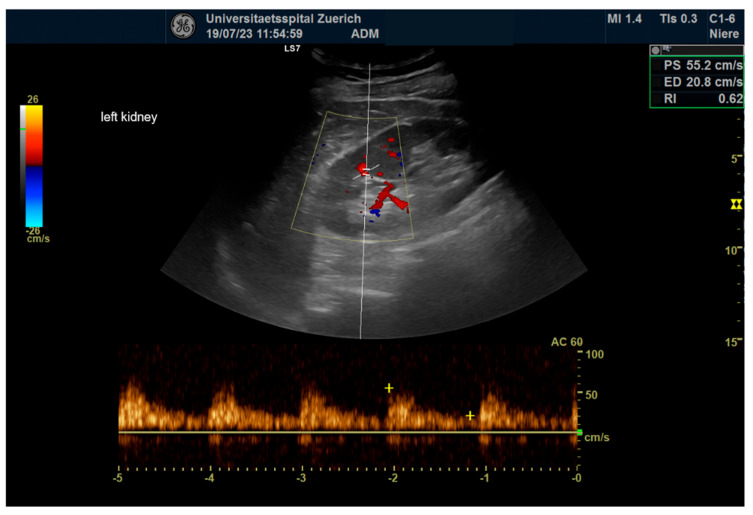
Color coded duplex sonography of renal resistance index values in renal ultrasound.

**Figure 5:**
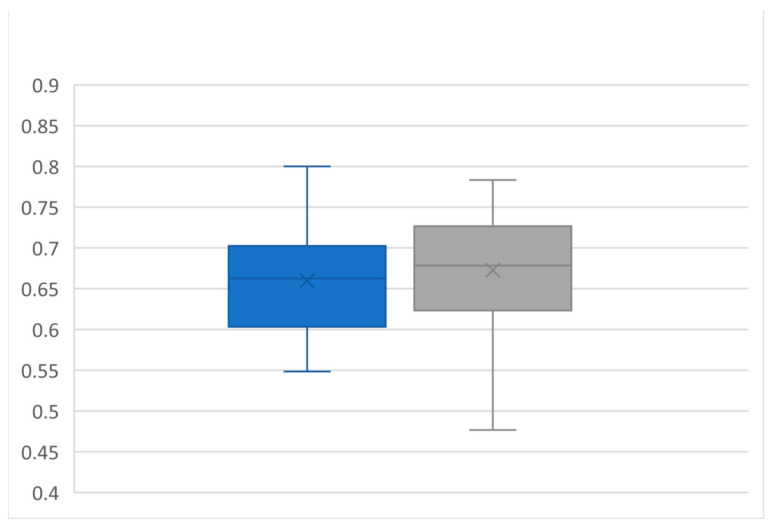
Boxplots of renal resistance index preoperative (blue boxplot) and postoperative (grey boxplot). All values are expressed as mean ± standard deviation: 0.66 ± 0.057 vs. 0.67 ± 0.067, *p* = ns, by paired two-sided students *t*-test.

For 64 kidneys in 32 consecutive patients, pre- and postinterventional examinations were carried out. Exemplary illustrations of renal B-mode pictures, renal arborization, and RRI measurements are displaced in Figure 2, Figure 3 and Figure 4. Sonomorphologically, the kidneys examined were inconspicuous, with a preoperative mean size of 107.4 × 52 mm and a mean parenchymal margin of 18.3 mm. The arborisation of the renal perfusion was preserved pre- and post-implantation. The RRI did not differ before and after implantation (0.66 ± 0.06 versus 0.67 ± 0.07; *p* = 0.10) (Figure 5). 

### 3.7. Renal Functional Outcomes

**Figure 6:**
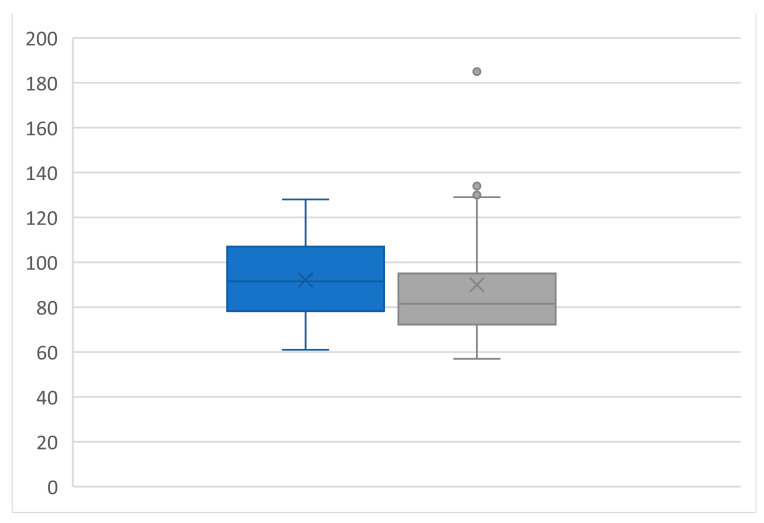
Boxplots of creatinine preoperative (blue boxplot) and postoperative (grey boxplot). All values are expressed as mean ± standard deviation: 92.1 ± 18.5 vs. 90.0 ± 28.2, *p* = ns, by paired two-sided students *t*-test.

**Figure 7:**
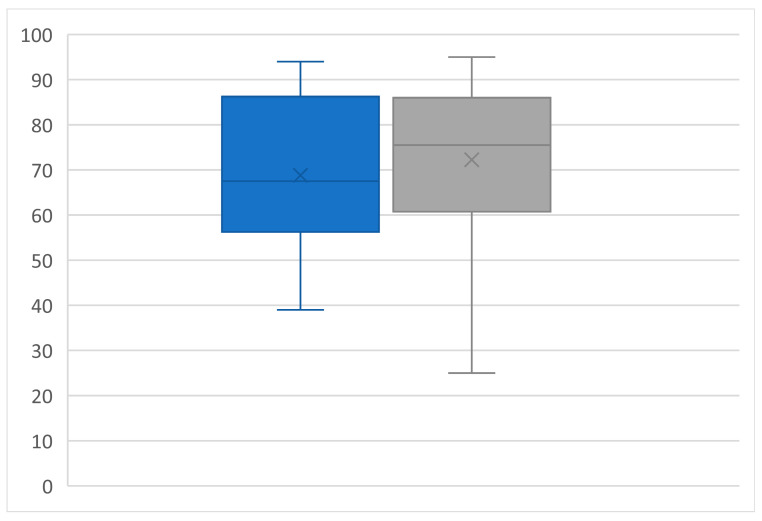
Boxplots of eGFR preoperative (blue boxplot) and postoperative (grey boxplot). All values are expressed as mean ± standard deviation: 68.8 ± 15.8 vs. 72.3 ± 18, *p* = ns, by paired two-sided students *t*-test.

Due to the in- and exclusion criteria, none of the patients experienced postoperative severe impairment of renal function. The mean creatinine value was 92.1 ± 18.5 µmol/L preoperatively and 90.0 ± 28.2 µmol/L postoperatively (*p* = ns). The mean eGFR was 68.8 ± 15.8 mL/min preoperatively and 72.3 ± 18.0 mL/min postoperatively (*p* = ns, Figure 6 and Figure 7). The ultrasound evaluation and renal function outcomes are shown in Table 4. 

### 3.8. Subgroup Analysis

We performed a subgroup analysis regarding the diameter and length of the implanted bridging stent grafts. The RRI did not differ before and after implantation of bridging stent grafts smaller or equal to the median diameter of all implanted bridging stent grafts (0.66 ± 0.05 vs. 0.67 ± 0.06, *p* = ns). In addition, the RRI also did not differ after implantation of a bridging stent graft smaller or equal to the median length (0.67 ± 0.05 vs. 0.68 ± 0.07, *p* = ns). Implantation of a bridging stent graft larger than the median diameter resulted in a significant increase in the RRI postoperatively (0.64 ± 0.07 vs. 0.68 ± 0.08, *p* = 0.0014). Implantation of a bridging stent graft larger than the median length did not result in a significant change in the RRI pre- and postoperatively (0.64 ± 0.07 vs. 0.65 ± 0.06, *p* = ns).

Comparison between the pre- and postoperative RRI in BEVAR and FEVAR showed a significant increase in the postoperative RRI after BEVAR (0.64 ± 0.06 vs. 0.66 ± 0.07, *p* = 0.03). In FEVAR procedures, there was no significant postoperative change of the RRI compared to preoperative (0.66 ± 0.05 vs. 0.67 ± 0.06, *p* = ns) (Table 5). 

## 4. Discussion

The major finding of our study is that bridging stent graft implantation in non-diseased renal arteries does not alter the RRI. The RRI therefore might be suitable for assessing renal perfusion after complex endovascular aortic therapy. 

In addition, with adequate perioperative nephroprotective management, e.g., avoiding dehydration, adequate hydration, and minimisation of contrast agent exposure, renal function did not decrease after the endovascular procedure. 

Theoretically, stent graft implantation into a renal artery induces permanent alteration of the physiological flow behavior. The deployment of a metal stent results in changes in vessel compliance with enhanced stiffness of the stent–arterial wall complex. This might induce alteration in compliance affecting the velocity of the blood flow alternating DUS measurements [29,30]. To our knowledge, this is the only study evaluating the influence of bridging stent graft implantation into non-diseased renal arteries on the RRI. A cohort study published in 2016 of 49 patients with 88 bridging stent graft implantations into renal arteries showed no significant differences in postoperative median renal artery PSV measurements after FEVAR compared to the baseline median PSV in a follow-up of two years. Therefore, the authors concluded that covered stent placement in non-stenotic renal arteries during FEVAR is safe and durable, with normal PSV in most patients. 

Interestingly, in this cohort, 17 renal arteries in 13 patients exceeded the ≥60% stenosis threshold for DUS criteria for native arteries, whereby an additional CTA showed no significant renal artery stenosis [31]. This finding suggests that duplex velocity criteria for stenosis in native renal arteries appeared to overestimate the severity of stenosis in renal artery-covered stents. However, this is in line with duplex velocity criteria for restenosis after carotid artery stenting with elevated flow velocity thresholds for in-stent restenosis compared to significant internal carotid artery stenosis in native arteries. Possible reasons for this could be—as discussed before—changes in the biomechanical properties of the artery, including higher stiffness of the arterial wall and decreased arterial compliance. This leads to a reduced alteration of the volume of the arterial segment during different phases of the pulse wave [32].

A sustainable postoperative PSV measurement in renal arteries is challenging in the majority of patients. Often, we experience a lack of ability to follow instructions to perform a proper examination. In addition, multiple physical factors like sonic shadowing due to bowel gas and obesity or increased abdominal fat, etc., may limit the DUS evaluability of renal arteries. Therefore, we focused our investigations on the more reliable assessable RRI.

Arterial stent graft implantation could result in hemodynamic effects in the end-organ, e.g., reduce the systolic and increase the diastolic velocity in the end organ. This may result in a decrease in the RRI. Interestingly, and in contrast to that theory, in our subgroup analysis comparing BEVAR with FEVAR procedures, bridging stent graft implantation into the renal arteries resulted in a statistically significant postoperative increase in RRI values. In this subgroup, the median length of the bridging stent graft was longer than in the total cohort (57 mm vs. 27 mm respectively). This might result in a higher rigidity of the stent graft–vessel wall complex with an increase in the PSV in the stent graft. This is in line with the findings by Heneghan et al. [31]. In 19% of the evaluated stented renal arteries, they reported a PSV suggesting a >60% stenosis in DUS, which could not be verified in CTA. Another hypothesis to explain this finding might be a decrease in the end-diastolic velocity in the stent graft caused by the higher rigidity of the vessel–wall complex and the therefore reduced or eliminated windkessel effect. However, whether this possible effect really has a clinical relevance to renal perfusion is unclear. 

Arterial stiffness may be defined as the resistance offered by vascular walls to deformation powered by a propulsive force, as reviewed by Bissacco et al. [33]. The authors reported that increased aortic stiffness with an increased pulsewave velocity (PWV) is associated with renal blood flow impairment due to a reduction in peripheral artery perfusion. Therefore, stent graft implantation into non-diseased renal arteries may induce high arterial stiffness of the vessel–stent complex. This is demonstrated in our cohort in an additional subgroup analysis after implantation of bridging stent graft with a diameter larger than the median diameter of 6 mm, leading to a statistically highly significant increase between pre- and postoperative RRI. This finding is surprising, because, in native renal arteries, the vessel diameter has not been reported to influence the RRI, but has been shown to influence renal artery compliance, which is reduced after stent graft implantation [34]. 

Several studies showed a tendency for higher branch instability and a lower patency rate for renal target arteries compared to visceral arteries. Fenestrated compared to branched repair seems to have similar patency rates [35,36,37]. This finding was also pointed out in a systematic review published in 2021, which showed a three times higher complication rate for renal vessels compared to visceral vessels, with a similar reintervention rate [16]. Mastracci et al. highlighted some theoretical postulations of possible reasons, e.g., anatomical factors such as the high variability of the renal angle and the smaller vessel diameter compared to the visceral vessels, as well as physiological aspects, such as the high respiratory motion of the renal arteries and the higher resistance of the end-organ [38]. Due to the risk of those potential target vessel complications, especially for renal arteries, adequate intraoperative imaging control, as well as short- and long-term follow-up imaging control, is mandatory. Intraoperatively, DSA is the method of choice not only to detect possible endoleaks, but also to verify correct stent graft deployment and the patency of the bridging stent grafts and target vessels [3]. However, it has been demonstrated that DSA alone does not detect all findings, leading to re-interventions like stent compression or kinking, arterial dissection, or thrombus compared to cone beam CT [18]. 

In addition, cone beam CT, as well as postoperative CTA, leads to a higher exposition of radiation and additional iodine contrast agent load, with their adverse effects [39]. MRI as a radiation-free imaging modality might be more sensitive in the detection of endoleaks compared to CTA in follow-up imaging; however, it currently cannot replace CTA for the imaging control of complex endovascular aortic repair regarding target artery complications because of the artifacts generated by the frame of the endoprosthesis and the bridging stent grafts themselves [40]. On the contrary, DUS, as well as contrast-enhanced ultrasonography (CEUS), has been shown to be a valid method for the control and follow-up after F/BEVAR when it comes to bridging stent graft patency [31,40]. However, despite being a widely available diagnostic tool, those methods have their limitations due to their known investigator dependence and time-consuming nature. 

### 4.1. Limitations 

Due to the strict application of exclusion criteria, we see an important limitation of our study: the relatively small sample size that could be evaluated in total. This is due to the goal of avoiding the impact of inadequate technical success or insufficient ultrasound evaluation. Approximately one-third of the possible eligible patients had to be excluded because of a lack of proper RRI measurement according to ESVS standard criteria. This might be surprisingly high but reflects the limitations of routine ultrasound examinations in hospitalized patients in an intensive care setting. With a mean age of 73, for 9 years the treated patients are prone to developing postoperative delirium, making it difficult—if not impossible—to follow specific instructions for the ultrasound examination.

### 4.2. Impact on Clinical Practice

To avoid possible bias on the RRI for a strict statistic and scientific evaluation, we excluded patients with renal artery stenosis, previous renal artery stenting or parallel graft implantation, retro-grade puncture of the renal artery via lumbotomy, perioperative renal artery dissection or occlusion, and postoperative acute renal failure. Therefore, moderate RRI changes in clinical patients might be detected in clinical patients after bridging stent graft implantation when those criteria are not strictly applied. 

If used in clinical routine follow-up examination, these strict exclusion criteria cannot be applied in the majority of patients if the RRI evaluation should be a standard procedure with six RRI measurements in both kidneys. 

## 5. Conclusions

The results of this study suggest that there is no relevant change in the RRI after successful bridging stent graft implantation in a non-diseased renal artery during FEVAR and/or BEVAR procedures. The RRI seems to be suitable for assessing renal perfusion after complex endovascular aortic therapy and can serve as an easily accessible method to detect target artery complications after complex endovascular aortic aneurysm repair. However, in clinical practice, there are limitations like the often reduced compliance of the patients, limiting physical factors, and the availability of qualified examiners and adequate ultrasound systems in the early postoperative phase. Further, mid- and long-term investigations are warranted to clarify the effects of bridging stent grafts in non-diseased renal arteries. The sources of possible bias and their possible impact on RRI measurements in clinical routine might be an especially interesting approach to be evaluated in a follow-up study.

## Figures and Tables

**Table 1:** Patient characteristics.

Demographics	*n* = 32	%
Age (years, mean ± SD)	73.9 ± 8.2	-
Male	27	84%
Female	5	16%
**Comorbidities**		
Hypertension	30	94%
Smoking	25	78%
Hypercholesterolemia	31	97%
Diabetes	4	12%
**Aneurysm characteristics**		
Juxtarenal	22	69%
Pararenal	2	6%
Suprarenal	0	0%
TAAA	8	25%
**Diameter (mm, mean, ± SD, range)**	62.8 ± 11.2 (44–100)	-

Baseline characteristics from 32 patients in whom successful bridging stent-graft implantation into non-stenosed renal arteries was performed. SD, standard deviation; TAAA, thoraco–abdominal aortic aneurysm.

**Table 2:** Treatment modalities.

	*n*	%
Hospitalization (days, mean ±SD)	9.5 ± 7.8	-
**Indication (*n* = 32)**		
Elective, primary	19	59%
Elective, secondary	11	34%
Urgent (symptomatic)	2	6%
**Type (*n* = 32)**		
FEVAR	26	81%
BEVAR	6	19%
**Endoprosthesis (*n* = 32)**		
Off-the-shelf	5	16%
CMD	23	72%
PMD	4	12%
**Bridging stent grafts (*n* = 64)**		
Balloon-expendable	63	98%
Self-expendable	1	2%
**Treated visceral arteries (*n* = 32)**		
CT	19	59%
SMA	27	84%

Treatment modalities from 32 patients in whom successful bridging stent-graft implantation into non-stenosed renal arteries was performed. SD, standard deviation; FEVAR, fenestrated endovascular aneurysm repair; BEVAR branched endovascular aneurysm repair; CMD, custom-made device; PMD, physician-modified device; CT, celiac trunk; SMA, superior mesenteric artery.

**Table 3:** Bridging stent graft details.

		BEVAR	FEVAR	Total
Product	Diameter/Length (mm)	*n*	*n*	*n*
**Advanta^®^**				
	6/22	0	12	12
	6/38	0	1	1
	8/32	0	2	2
**Bentley^®^**				
	7/23	0	1	1
**E-ventus^®^**				
	6/22	0	5	5
	6/58	1	0	1
	8/37	1	0	1
	8/57	1	0	1
**iCover^®^**				
	6/17	0	1	1
	6/27	0	25	25
	6/37	0	2	2
	6/57	8	0	8
	8/27	0	2	2
	8/37	1	2	3
**Viabahn^®^**				
	6/50	1	0	1
**Total (*n*)**		13	53	66
**Median diameter (mm)**		6	6	6
**Median length (mm)**		57	27	27

Applied bridging stent-grafts in branched vs. fenestrated procedures. FEVAR (fenestrated endovascular aneurysm repair), BEVAR (branched endovascular aneurysm repair), Note: there are two cases with implantation of two bridging stent grafts in one renal artery (one right and one left; different cases).

**Table 4:** Results.

Renal Parameters (*n* = 64)	Pre-Operative	Post-Operative	*p*
RRI	0.66 ± 0.06	0.67 ± 0.07	ns
Length (mm)	107.1± 13.8	107.9 ± 14.5	ns
Wide (mm)	52.4± 7.9	52.9 ± 7.8	ns
Parenchymal margin (mm)	18.3 ± 3.2	18.6 ± 2.8	ns
**Laboratory Results (*n* = 32)**			
Creatinine (µmol/L)	92.1 ± 18.5	90.0 ± 28.2	ns
eGFR (ml/min)	68.8 ± 15.8	72.3 ± 18.0	ns

Results from 32 patients in whom successful bridging stent-graft implantation into non stenosed renal arteries was performed. Values are expressed as mean ± standard deviation. Values were calculated with the use of a paired two-sided student’s *t*-test. Statistical significance was assumed if *p* < 0.05. RRI, renal resistance index; eGFR, estimated glomerular filtration rate; ns, non-significant. Parameters are given as mean ± standard deviation.

**Table 5:** Results subgroup analysis.

Bridging Stent Graft	RRI Pre-Operative	RRI Post-Operative	*p*
Diameter ≤ Median (6 mm), *n* =55	0.66 ± 0.05	0.67 ± 0.06	ns
Diameter > Median (6 mm), *n* = 9	0.64 ± 0.07	0.68 ± 0.08	0.0014 *
Length ≤ Median (27 mm), *n* = 44	0.67 ± 0.05	0.68 ± 0.07	ns
Length > Median (27 mm), *n* = 20	0.64 ± 0.07	0.65 ± 0.06	ns
**Procedure**			
BEVAR, *n* = 12	0.64 ± 0.06	0.66 ± 0.07	0.03 *
FEVAR, *n*= 52	0.66 ± 0.05	0.67 ± 0.06	ns

Values are expressed as mean ± standard deviation. Values were calculated with the use of a paired two-sided student’s *t*-test. Statistical significance was assumed if *p* < 0.05. ns, not significant; FEVAR (fenestrated endovascular aneurysm repair), BEVAR (branched endovascular aneurysm repair) * *p* < 0.05 = significant; numbers are given as mean ± standard deviation.

## Data Availability

The data presented in this study are available on request from the corresponding author, the data are not publicly available due to ethical restrictions.
